# Toward a Comprehensive Approach to the Collection and Analysis of Pica Substances, with Emphasis on Geophagic Materials

**DOI:** 10.1371/journal.pone.0003147

**Published:** 2008-09-05

**Authors:** Sera L. Young, M. Jeffrey Wilson, Dennis Miller, Stephen Hillier

**Affiliations:** 1 School of Public Health, University of California, Berkeley, California, United States of America; 2 Department of Pediatrics, University of California Davis, Davis, California, United States of America; 3 Soils Group, Macaulay Institute, Craigiebuckler, Aberdeen, Scotland, United Kingdom; 4 Department of Food Sciences, Cornell University, Ithaca, New York, United States of America; University of Toronto, Canada

## Abstract

**Background:**

Pica, the craving and subsequent consumption of non-food substances such as earth, charcoal, and raw starch, has been an enigma for more than 2000 years. Currently, there are little available data for testing major hypotheses about pica because of methodological limitations and lack of attention to the problem.

**Methodology:**

In this paper we critically review procedures and guidelines for interviews and sample collection that are appropriate for a wide variety of pica substances. In addition, we outline methodologies for the physical, mineralogical, and chemical characterization of these substances, with particular focus on geophagic soils and clays. Many of these methods are standard procedures in anthropological, soil, or nutritional sciences, but have rarely or never been applied to the study of pica.

**Principal Findings:**

Physical properties of geophagic materials including color, particle size distribution, consistency and dispersion/flocculation (coagulation) should be assessed by appropriate methods. Quantitative mineralogical analyses by X-ray diffraction should be made on bulk material as well as on separated clay fractions, and the various clay minerals should be characterized by a variety of supplementary tests. Concentrations of minerals should be determined using X-ray fluorescence for non-food substances and inductively coupled plasma–atomic emission spectroscopy for food-like substances. pH, salt content, cation exchange capacity, organic carbon content and labile forms of iron oxide should also be determined. Finally, analyses relating to biological interactions are recommended, including determination of the bioavailability of nutrients and other bioactive components from pica substances, as well as their detoxification capacities and parasitological profiles.

**Significance:**

This is the first review of appropriate methodologies for the study of human pica. The comprehensive and multi-disciplinary approach to the collection and analysis of pica substances detailed here is a necessary preliminary step to understanding the nutritional enigma of non-food consumption.

## Introduction

Pica, the craving and subsequent consumption of non-food substances such as earth, charcoal, uncooked rice, starch, and ice, has been an enigma since it was first documented by Hippocrates in the 4^th^ century BC [1]. Although pica is widespread and associated with serious health problems, neither its causes nor its consequences are clearly understood.

There are many reasons for our poor understanding of pica. These include the lack of awareness of pica by researchers, the concealment of pica by those who practice it, biases and judgmental nature of those who study it, the assumption that pica is a mental illness, and research designs that are incapable of answering questions of causality [2]. Furthermore, pica is a complex behavior that requires understanding of cultural attitudes, physiology, biochemistry, and soil science. Pica research thus requires a multidisciplinary approach. However, the research approaches frequently used by those who have studied it have been limited to their own particular specialty. Nutritionists have discussed dietary issues, cultural anthropologists have been concerned with cultural transference, geographers have focused upon the characteristics of geophagic soils, and parasitologists have studied the nematode content of pica substances. This diffusion of effort within a spectrum of different objectives has led to irregular sampling and uneven, incomplete analyses of data related to pica.

The purpose of this paper is to encourage a multidisciplinary approach to the study of pica by describing applicable procedures and methodologies from a wide range of disciplines. Some procedures for studying geophagy (earth-eating) in animals have been previously outlined by Mahaney and Krishnamani [3]. In this paper, however, we aim to suggest a more comprehensive approach that is directed to the study of pica in humans and, while focusing on geophagy, expand beyond it to encompass the study of a variety of other pica substances. We provide both a critical overview of methods used by the many disciplines interested in pica as well as suggest techniques not previously used in its study. This paper is also novel in that it outlines how each method may be applied to the testing of the various hypotheses about pica.

It is our hope that this paper will facilitate the standardization of data collection and analysis. Once such data is appropriately collected, more uniform data sets can be used to finally test the many hypotheses about pica.

### What is pica?

Pica is typically defined in scientific communities as “the persistent eating of non-nutritive substances” [4] or “the tendency or craving to eat substances other than normal foodstuffs” [5]. Both of these definitions have serious limitations. The term “non-nutritive” is problematic because nutrients can be obtained from some pica substances (e.g. starch is high in calories), and it is possible that micronutrients can be obtained from soils. The phrase “normal foodstuffs” is ambiguous because normalcy is distinctly culturally determined. Finally, neither definition mentions the strong desire for pica substances that most who engage in pica experience.

Geophagy (or geophagia) is the most common type of pica described in the literature, although many other substances have been characterized as pica including baby powder, chalk, ash, ceramics, paper, paint chips, charcoal, and large quantities of ice [6], [7]. Amylophagy (or amylophagia, the consumption of uncooked starch) is the second most commonly described pica phenomenon. Corn starch is the most typically consumed form of uncooked starch, but reports of the consumption of raw wheat flour, laundry starch and uncooked rice have also been classified as amylophagy [8]–[12].

For the purposes of describing analytical methods, we have grouped pica substances into uncooked food and non-food substances (Table 1). This division is heuristic; pica consumers do not typically make such distinctions. While it is possible that the consumption of earth is a different phenomenon than the consumption of other non-food substances, four observations support a commonality.

**Table 1:** Typical pica substances.

Uncooked food substances	Non-food substances
Corn starch	Ash
	Baby powder
Flour	Chalk
	Charcoal
Ice (ice and freezer frost)	Earth
	Pottery
Uncooked rice	Plaster

1. Those who eat earth are frequently consumers of other non-food substances [e.g. 13], [14].
2. Those who consume the more manufactured substances state that they use them as a replacement for earth, either because the desired soil is unavailable or socially unacceptable [e.g. 15]–[18].
3. Except for ice, most pica substances are absorptive in the dry state (e.g. charcoal, ash, clay, ground uncooked rice) and all readily absorb moisture.
4. Pica substances are typically craved with great intensity or “devouring passion” [19]. For example, one clay vendor in Johannesburg said that her “customers go crazy without the stuff” [20]. A woman in the Southern United States explained, “I used to tear up a bank. (…) I went wild over it, I ate so much. I was killin' that dirt.” [e.g. 21]. Women in Zanzibar use the term “kileo”, which is also the term for drug addiction and alcoholism, to describe their feelings for pica substances [22].

Because of this evidence of commonality, we believe non-food cravings cannot be fully understood by focusing solely on geophagy and therefore strongly encourage researchers to study other pica substances as well.

### Pica hypotheses

There are three major groups of hypotheses about the physiological causes of pica: hunger, micronutrient deficiency, and protection from toxins and pathogens (Young S, Sherman PW, Lucks J, Pelto G, (in preparation)). It has also long been hypothesized that pica causes micronutrient deficiencies, namely anemia [23]. Yet only a handful of studies addressed the question of whether the consumption of these substances is motivated by even one of these possible causes, and even fewer have studied the health effects of practicing pica. No study has comprehensively tested all hypotheses for any pica substance.

The *hunger hypothesis* posits that people consume non-food substances because they do not have anything else to eat [24], [25].

The *micronutrient deficiency (nutritional) hypothesis* posits that people eat non-food substances because they are deficient in iron, zinc, calcium, or some other micronutrient and that pica is an attempt to increase micronutrient intake [26]–[28]. Another version of this hypothesis is that a micronutrient deficiency causes disturbed taste sensitivities or malfunctioning appetite-regulating brain enzymes which causes non-food substances to become appealing [29]–[32]. In this scenario, pica is a consequence of micronutrient deficiency, but not an attempt to remedy it.

The *protection hypothesis* states that pica is motivated by an attempt to mitigate the harmful effects of plant chemicals or microbes [33]–[36]. It is proposed that pica substances protect by either adsorbing pathogens and toxins within the gut lumen or coating the surface of the intestinal endothelium, thereby rendering it less permeable to toxins and pathogens. According to this hypothesis, overt gastrointestinal distress, which can be the result of exposure to either toxins or pathogens [37], [38], would also trigger pica. Additionally, this hypothesis implies that pica substances would be ingested during periods of rapid growth, i.e. the times of greatest need for protection from toxins and microbes. Under this hypothesis, childhood and pregnancy, especially early pregnancy (which is the critical period of organogenesis [39], [40]), would be the periods when pica was most likely to occur. Pregnant women, who are immunologically suppressed [41], [42], may also need protection from substances that would normally be harmless. Although the mechanisms by which this could occur are not well elucidated, increased sensitivity to pathogens and toxins may occur in early pregnancy when levels of estradiol, which triggers nausea, are highest [43].

Both clay and raw starch have been shown to be effective in this respect [44]–[47]. Clay is a well-established treatment for gastrointestinal distress. For example, Kaopectate®, a widely used over-the-counter treatment for nausea, diarrhea and vomiting, takes its name from kaolin, the clay that was formerly the active ingredient. Starch has not been used in clinical settings to treat gastrointestinal distress, but it has been shown to adsorb poisons and pathogens that cause gastrointestinal distress [48].

A handful of scientists have suggested that pica is a “protective” response to psychological stress [49]–[53]. Because most of these studies were individual case studies or stress was measured in non-standardized ways, more exploratory research is needed before the hypothesis that pica is a response to stress merits high research priority.

A fourth posited relationship is that pica causes anemia [23]. In this scenario, the cause of pica is not known, but the consequence is said to be anemia. This may happen if pica substances inhibit the absorption of dietary nutrients required for the production of hemoglobin (namely iron or zinc) [36], [54], [55]. Pica could also cause anemia if it is a vector for nematode infections. In fact, one of the oldest allegations leveled at pica, especially geophagia, is that it is a risk factor for the transmission of parasitic nematodes, namely *Ascaris lumbricoides* (roundworm), *Trichuris trichiura* (whipworm), *Toxocara* spp. and hookworms [15], [24], [56]–[60].

The preceding four explanations deal with the functional significance of pica. Another group of explanations attribute pica to “culture” in a broad sense. Early writers blamed “culture” for pica, attributing “this perversion of taste” to “the tenacity of ignorance…characteristic of colonial subjects” [61]. Of late, others have elucidated rich cultural meanings of pica, especially geophagy, within the context of particular cultures [62]–[65]. Thus, it has been suggested that it “makes sense” that women eat earth because their traditional roles in some societies as potters and gardeners bring them close to the soil [66] or that the fecundity of the earth makes it appropriate for ingestion because of “the cultural associations of soil-eating with blood, fertility and femininity” [67, p. 1078].

Pica is undoubtedly a practice affected by cultural norms [52], [68]. However, in this paper we have opted to focus on methods to study the physiological underpinnings and ecological forces of pica, which to some extent underlie cultural manifestations. A focus on physiology does not preclude attention to cultural factors. For example, it is important to understand why some cultures sanction pica while others do not. It may also be valuable to study the persistence of human pica in settings where it is not culturally sanctioned. For ethnographic techniques that have been applied in the study of the cultural study of pica, we refer the reader to the above articles that focused on the cultural dimensions of pica [52], [68] as well as Bernard [69].

## Methods

This paper is based on a review of the literature, the authors' own training in the anthropological, food, soil and nutritional sciences, and experiences with the study of pica. We suggest an approach based on six different procedures and methodologies, namely 1) Oral interview, 2) Sample collection, 3) Physical analysis 4) Mineralogy 5) Chemical analysis, and 6) Biological interactions. Table 2 indicates which of the various procedures and methodologies is applicable to the testing of each hypothesis.

**Table 2:** Relationship of procedures and methodologies to hypotheses.

Hypotheses		Procedures and Methodologies					
		(1) Oral interview	(2) Sample collection	(3) Physical analyses	(4) Mineralogical analyses	(5) Chemical analyses	(6) Biological interactions
Causes:	Hunger	X		X	X		X
	Micronutrient deficiency	X	X	X	X	X	X
	Protection	X	X	X	X	X	X
Consequence:	Anemia		X	X	X	X	X

## Results

### 1. Oral interview

A thoughtful conversation with the consumer and, when possible, the producer of pica substances, is the first step in obtaining an accurate understanding of pica practices. In the course of this communication, it is imperative to avoid judgmental behavior, comments, or questions, and to conduct interviews in a tolerant and compassionate way.

In our fieldwork in Zanzibar, Tanzania, researcher training included an explanation of how people around the world have eaten non-food substances for thousands of years and that scientists still did not know if pica has health benefits. We emphasized that it was for this reason that this research was being conducted. In their interviews with people who engaged in pica, researchers were taught to emphasize that there were no right or wrong answers to questions and that the interviewee was the pica expert. They learned to foster a spirit of teamwork with the respondents and to emphasize that everyone would be working together to help solve this mystery. A week of training and practice interviews took place before any fieldwork began.

Specific questions that should be asked in the oral interview, together with their underlying rationale, are as follows:

#### Question 1. *What are the substances that you have heard that people like to eat that are not normal food?*

A general question about the consumption of items that are not typically thought of as food *by other people* is an excellent way to begin an interview. First of all, it permits the respondent to warm up to the topic. Establishing that other people in the community eat these substances may help the respondent to feel less embarrassed about his or her own pica behavior. Finally, this list can be revisited at the end of the interview, to confirm that the respondent has listed all pica substances he or she consumes.

#### Question 2. *What is the local name, brand name, or type of pica substance desired or consumed?*

This will help others to know if this substance has already been studied and assist interested researchers in obtaining subsequent samples at a later date. Furthermore, different manufactured products may contain different materials, e.g. Crayola chalkboard chalk contains slightly different ingredients from other brands. Similarly, the consequences of toilet tissue paper consumption [32] are different from those of eating pages of a novel [70]; information would be lost if the substance was simply described as paper. For these reasons, the substance consumed should be described in as much detail and as accurately as possible.

#### Question 3. *How much does the craved substance cost?*

The absolute cost in local currency is less important than the cost relative to the individual's resources. In her interviews of Midwestern American women, Cooksey learned that low-income women were willing, and even compelled, to spend large amounts of money daily on purchasing multiple “party bags” of the specific brand of ice they craved [71]. The amount of money people are willing to spend to obtain pica substances is indicative of the strength of their desire as well as the degree to which their cravings impact their daily lives and the lives of their families. Ideally, the cost of pica substances would be expressed as a percentage of the food budget.

#### Question 4. *Where does the substance craved come from?*

While the source of some pica substances, like Johnson's® baby powder or Argo® cornstarch are obvious, other materials like earth, charcoal and ice can come from many places. Thus, specific questions should be asked about the source. If the consumer does not know the origin of the substance, it may be possible to pursue the substance's origin by visiting the person who produced it or the shop that sold it.

Asking the consumer if others obtain pica material from the same cave, market stall, riverbed, charcoal heap, etc., can be a good way to find more informants (termed a “snowball sample” [69]). It will also provide an indication of the prevalence of pica behavior in relation to a particular source. Furthermore, it may lead to the discovery of non-human consumers of the same substance, as it did in Zanzibar (SLY field notes, 7/2006) and Zambia [72].

**Figure 1:**
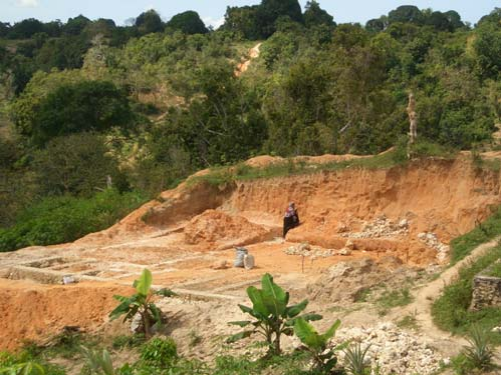
The context of the source of pica samples should be documented with photography, such as this soil sample from a building site in Zanzibar, Tanzania.

If possible, photograph the source, capturing as much of the setting as possible (Figure 1). The photos will not only illuminate the subject matter, but may contain important information (e.g. geological data, proximity to pollutants) that is easy to overlook during the interview (cf. Figure 1). Noting GPS coordinates will facilitate subsequent returns to the site.

#### Question 5. *How is the pica substance prepared before consumption?*

Pica consumers frequently prepare raw pica materials by sifting, grinding, pan frying, baking, or moistening the material. Pica substances that are sold in markets have typically already undergone an elaborate production process [e.g. 73], [74].

Preparation has consequences for the physical, chemical, and parasitological properties of the substance. For example, grinding the substance [e.g. 75] can greatly increase its pH (cf. *Measurement of pH* below). Thoroughly heating the substance [e.g. 76] can reduce the viable geohelminth content. The fact that red fragments of clay are reportedly manually removed from geophagic clay [e.g. 73] may be important with respect to the iron content of the bulk material. A thorough description of preparation techniques will provide valuable information about the plausibility of potential consequences.

#### Question 6. *How is the pica substance stored?*

Storage of the substance may give information relevant to the parasitological and microbiological profile of the material. If clay is stored in a moist environment, it can easily maintain viable hookworm eggs or harbor potentially harmful fungus. If it is not stored at all, this could indicate immediate availability or minimal preparation. If the substance is hidden away, this could be indicative of societal attitudes. The description of the storage by one Louisiana woman indicates how unacceptable this behavior was: “I keep it in a coffee can… but I would hide it. I'd keep it where people couldn't see it. I'd keep the can in a bag” [76, p. 66]

#### Question 7. *Why do you eat this material? What makes it appealing?*

An obvious way to determine why people like to eat earth, charcoal, ash, and so forth, is to ask them. Yet this seemingly straightforward question is frequently difficult and often impossible to answer. For example, when this question was asked of pica consumers in Zanzibar, the overwhelming response was that they “don't know”, that they “just do”, or a tautological answer such as “I like to eat it because I crave it” [22]. Other researchers have experienced similar difficulties [e.g. 76]. It seems that the only motivations that can be readily explained are hunger and heartburn, but these instances are rare [e.g. 77].

There may be a number of reasons why respondents struggle to answer this question. The consumer may have never tried to articulate an answer, they may be too embarrassed to discuss their love of dirt, or they may really not understand their desire. One way to help the consumer to answer this question is to ask about the particular features of the substances they like, such as smell, flavor, color, temperature, texture, or even the memories it conjures.

#### Question 8. *What are the age, gender, and other relevant health details of consumer?*

Most hypotheses about the etiology of pica are related to physiological status (e.g. childhood, pregnancy, anemia, hunger). For example, pica has come to be associated with pregnancy so strongly that the craving for earth may even be regarded as a sign of pregnancy. One senior government physician said, “It would be very surprising if pregnant women in Malawi did not eat clay. That's how you know when you are pregnant!” [72]. It has similarly been equated with anemia. David Livingston, the famous explorer of East Africa, mis-translated the Swahili word for anemia as “the disease of earth-eating” [78, p. 346, 376].

While such remarks are indicative of patterns of association, it is important to be able to link the behavior of individuals at specific points in their lives to particular substances that can then be analyzed. Pointed questions about what the person was experiencing at the time of pica consumption, e.g. nausea, pregnancy, “no special time”, or pallor (indicative of anemia), permit these links to be made. While such questioning about health and life stage details is not a substitute for an epidemiological study, it will generate preliminary data that can help shape eventual large-scale surveys.

#### Question 9. *What other non-food substances do you crave? Are these used as substitutes or under different circumstances?*

If other non-food substances are craved, repeat the above questions. If the respondent initially says there are no others, prompt them using the list of substances generated in question 1.

Earth is typically the first pica substance that observers hear about or notice, usually because it is so strikingly *not* food. However, it may be easier for respondents if the interview does not commence by inquiring about earth, but about more food-like substances, such as starch or ice. After rapport is established, respondents may feel more comfortable to talk about their more “extreme” cravings. such as earth or plaster.

If more than one pica substance is consumed, elucidating the circumstances during which each substance is used can indicate similar or divergent properties. For example, slaves reported eating plaster, mortar, and coals when they were prevented from eating clay [79]. Several Zanzibari women reported eating clay when pregnant and uncooked rice when not pregnant or earth when they could not afford to purchase rice (SLY field notes 8/2006).

### 2. Sample collection

Results of analyses are only as good as the samples on which they are performed. Sample collection must therefore be conducted both carefully and systematically.

#### 1. *Samples of pica substances should be as similar as possible to what the person actually eats*

Identifying the substance that is eaten is only part of the collection process, as it may be prepared further before consumption. For example, at a hospital in North Carolina, “a big birthday celebration with her [a patient] family was planned, and on the menu was red Georgia clay, baked and topped with butter and salt” [80]. In Mississippi, in the 1970s, women often baked the earth they ate in an oven or a chimney, and some flavored it with vinegar and salt [81]. Charcoal is eaten directly or dissolved in hot water, to make a kind of “soot tea” [82]. In Zanzibar, the paper on cigarette butts is peeled off prior to consumption.

Observing the person prepare the material is a good way to corroborate if they do indeed prepare the material as they explained during the oral interview. It is also a good opportunity to probe further about their motivations for these behaviors.

#### 2. *Establish and collect the precise amount of pica substance consumed*

Estimations of ingestion amounts have been one of the most problematic aspects of quantitative analyses of pica substances. In studies of geophagic clays, especially those estimating the contribution of micronutrients to the diet, researchers have often made calculations based on clay intake reported by another study, even if that study took place on a different continent, several decades in the past, or in a different age group [26], [83].

There are several ways of establishing a more accurate measure of quantity consumed. Some researchers have asked informants to demonstrate how much they ate using pre-collected soil if it was not possible to have pica substances at the interview [84], [85]. A similar approach may be used with starch, uncooked rice, etc. It is also possible to ask respondents to estimate their consumption by using a locally familiar measurement, such as a teacup, handful, or ice cube tray.

The amount of earth consumed can also be measured indirectly. Because silica is not absorbed by the human gut, the silica content of stool can be an index of geophagy [86]. In several studies, the silica content of stool was measured to see how closely reported geophagy was tracked by stool silica content [84], [85], [87]. However, although this method seemed to be useful for identifying geophagous populations, it was not appropriate for quantifying individual soil intake because of the dependency of the silica content of the stool on the time since ingestion and the silica content of the soil consumed [84]. If rapport can be established in the interview, it is easier and more reliable to simply ask about earth consumption than to make calculations based on stool silica.

The most precise way to determine quantities consumed is for the consumer to measure out the quantity of the exact substance they consume over the course of a set period of time (day, week, etc.). For this procedure, once the substance is prepared, the consumer should be asked to put precisely the amount they consume in a sealable container [84]. Polyethylene plastic bags, such as Ziploc® brand bags, work well. This amount consumed should be for a specified period of time to achieve the greatest accuracy, e.g. one of the four doses eaten per day, the amount eaten in a single day, week, etc. Ideally, all consumers would use the same unit of time for their precisely measured sample. Because this is unlikely, careful note must be made of the period of time this sample represents. The air-dried sample should then be weighed to the nearest 0.1 g, and reports of amounts ingested should always state it as the air-dried weight. If the samples have been collected in very humid or moist conditions, it may be necessary to wait until returning to a laboratory to determine air-dried weights.

#### 3. *Collect 100 g of the pica substance for analysis*

The battery of analyses outlined below requires a significant amount of sample. One hundred grams may be much more than the participant is accustomed to collecting, and sometimes the consumer may not be willing or able to prepare such a quantity of the material. Too much work may be involved, e.g. grinding that much material may take hours, or the preferred type of charcoal is not available at the kitchen hearth at that moment. In these cases, it can be noted that the material is typically ground to a fine powder, with the grinding procedure done at a later stage. Alternatively, arrangements can be made to return later to collect more of the substance once it has accumulated or been prepared.

#### 4. *Place 10 g of prepared substance in plastic tube with formalin*

The substance should be placed in 10% formalin within 24 hours of collection in order to preserve all nematode stages for subsequent microscope analyses [88]. Falcon™ Tubes with screw caps have worked well for storage. Plastic gloves and caution should be used when handling formalin, as exposure can result in irritation, bums, and allergic reactions. Plastic tubes should then be placed in durable polyethylene sealable bags to contain any leakage.

#### 5. *Collect a control sample*

Much importance has been placed on collecting control samples in studies of geophagic earth in animals [3]. While it is very difficult to know that animals do not eat the control substance (since the absence of evidence cannot function as proof), with humans it is possible to ask which similar substance they would not eat. Their selection of rejected substances should be probed with questions such as “Why would you eat this uncooked rice but not this?” [88].

This exercise was illuminating in Zanzibar, where it was learned that the perceived cleanliness of the areas selected for geophagic earth was important and that finer charcoal particles were preferred over larger wooden chunks [88]. Finer charcoal was preferred because it was “softer”. The rejected earth samples were chosen from areas that were considered “contaminated”, as they came from areas where humans and animals could tread.

#### 6. *Archive the samples*

Each sample should be numbered, and these numbers, together with a few descriptive identifiers (e.g. sample type, date collected, place collected) should be recorded in a logbook. The sample number can then be hyphenated to distinguish each type of sample, e.g. “123-E” can refer to the exact amount normally consumed, “123-A” can refer to the sample for analyses, “123-F” for sample in formalin, and “123-C” for the control sample.

Carefully preparing durable labels is critically important. Writing with permanent marker on the outside of the container is necessary, but not sufficient. Labels smear, wear off and fade, especially when written directly on plastic. Writing on cloth tape, instead of directly on the plastic, reduces the risk of wear. Even with that precaution, a label should also be placed inside the sample bags. Both labels should contain, at minimum, the sample number and type (563-A, 563-C, etc.), the sample name, date collected and site from which it was collected.

**Figure 2:**
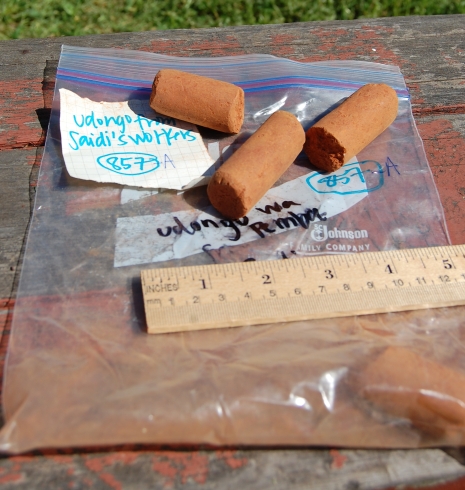
A close-up photograph of the sample in which the sample number is also visible is highly recommended. Ideally, a ruler should be included in the picture for scale.

The samples should also be visually recorded. A close-up photograph of the sample in which the sample number is also visible is highly recommended. Ideally, a ruler should be included in the picture for scale (Figure 2).

#### 7. *Shipping the samples*

If it is not possible to conduct analyses in the country or even region where the pica substances were collected, then it will almost certainly be necessary to obtain permits to ship the samples to a place where they can be analyzed. Import permits should be secured before collecting samples, and while in the country issuing the permission. Obtaining permission from the appropriate organization, e.g. United States Department of Agriculture or Department for Environment, Food and Rural Affairs in the UK, can take a long time and is more easily obtained if collaborating with a research institution that frequently imports soils for scientific purposes.

The regulations for shipping soil samples can be extremely strict. One US Customs and Border Patrol Agricultural Specialist suggested unused paint cans as ideal containers for transporting soil samples (Dr. Michael Berney, pers. comm.). They are durable and easy to open and reclose should officials want to inspect the contents. Other pica samples, like uncooked rice or baby powder, may be unproblematic for customs regulations.

### 3. Physical analyses

#### 1. Color

Before any analyses are done that could alter the pica substance, its color should be objectively established in the natural state, both before and after preparation. This is best done with the handy and easy-to-use Munsell color chart. The Munsell color chart describes colors in order of their hue (actual color), value (degree of lightness), and chroma (strength of color) and has found special use in the descriptive study of soils. For example, 7.5R 7/2 describes a reddish color (7.5R), of light value (7) and weak chroma (2).

In soils, color can be quite diagnostic of the predominant form of iron oxide. Thus, soils where the main iron oxide mineral is hematite (Fe_2_O_3_) are often a deep red (5YR), whereas soils where goethite [FeO(OH)] is the main iron oxide mineral are yellowish brown (2.5YR–7.5YR). Soils containing lepidocrocite, another form of iron oxyhydroxide, are a distinctive orange color (7.5YR).

#### 2. Particle Size Distribution Analysis (PSDA)

Soils normally consist of a mixture of inorganic mineral material, which is usually predominant, and organic material (also known as humus). The inorganic part of soil is comprised of several particle size fractions which have been classified according to a number of systems [89]. The two most common are the US Department of Agriculture and the International Union of Soil Sciences. The USDA scheme ranks particle sizes from medium to very coarse sand (0.25–2 mm in diameter), very fine to fine sand (0.05–0.25 mm), silt (0.002–0.05 mm) and clay (<0.002 mm). In the International scheme the medium to coarse sand fraction is (0.2–2 mm), fine sand (0.02–0.20 mm), silt (0.002–0.02 mm) and clay (<0.002 mm).

Before mineral samples can be further physically analyzed, soils need to be separated into these constituent particle size fractions through a process of disaggregation and dispersion. Determination of the relative proportions of each of these fractions is fundamental to the characterization of geophagic materials, for it tells us if the soil is clayey or sandy. This information is relevant to both the nutritional and protective hypotheses.

Disaggregation and dispersion of soils should first be attempted by purely physical means, using an ultrasonic probe and deionized water. For many soils and clays, such a treatment is sufficient to disaggregate them into their primary size fractions. The efficiency of the disaggregation process can be checked by microscopic examination of sand and silt fractions. Such an examination will clearly reveal the extent to which these fractions consist of discrete separated mineral grains as opposed to soil aggregates. Flocculation may prevent such a separation in some soils, but this can usually be overcome by the addition of a few drops of an alkaline reagent such as Calgon (sodium metaphosphate) in dilute concentration. It may also be necessary to remove organic matter or fine-grained iron oxides, both of which may act as cementing agents, in order to prevent particulate dispersion. Organic matter is usually removed by treatment with 30% hydrogen peroxide, and iron oxides by a sodium dithionite-citrate procedure. Both procedures are fully described in standard handbooks such as that published by the American Society of Agronomy [90].

Although there are various ways of determining the particle size distribution of soils and clays once the sample has been separated into its constituent fractions, they all have a similar goal: the quantification of the percentage of the sample that is sand, silt, and clay.

Particle size distribution analysis (PSDA) is usually done by a combination of sedimentation (using the pipette or hydrometer methods) and sieving. The detailed procedures are fully described by Day [91]. However, the use of laser diffraction for PSDA is becoming more widespread and is considered easy to use and capable of analyzing a broad range of particle sizes. In this method, the laser beam is transmitted through a cloud of particles of the material to be analyzed, scattered onto a Fourier lens which then focuses the scattered light onto a detector array. From the collected diffracted light data, a PSDA is inferred which has been found to be reasonably comparable to the results obtained by traditional PSDA methods. However, laser diffraction does consistently underestimate the clay fraction due to the platy form of the clay minerals. For example, it has been found that the <0.002 mm fraction defined by the pipette method corresponds with a grain size of 0.008 mm by laser diffraction [92]. Awareness of this problem enables suitable corrections to be made.

Given certain assumptions, determination of the amount of clay mineral in a bulk soil can also be done by X-ray diffraction. This method is based upon the quantitative analysis of the non-clay minerals using a reference intensity ratio method, combined with measurement of the 020 reflection for phyllosilicate minerals which is assumed to correspond to the amount of clay mineral present [93]. This assumption is justified for many soils, but breaks down if the soil contains significant amounts of micaceous minerals in the non-clay fractions.

#### 3. Consistency

The consistency or plasticity of geophagic soils and clays has long been noted as important to the attractiveness to the consumer. Preferred clays have been described as “unctuous” [e.g. 94], “smooth” [e.g. 95] and “greasy” [e.g. 96]. The plasticity will indicate if this quality is a commonality among geophagic samples. In soils, this property is defined by the Atterberg limits [97]. The liquid limit is the point at which a given amount of water added to a soil or clay converts it to a semi-fluid state; the plastic limit refers to the water content at which soil begins to crumble on being rolled into a 3 mm diameter thread. The plasticity index is the difference between the liquid and plastic limits and is a measure of the plasticity or consistence of the material. Standard methods for determining liquid/plastic limits are fully described by Sowers [97].

#### 4. Dispersion/flocculation characteristics

The dispersion/flocculation characteristics of pica materials may be an important physical property to determine, bearing in mind the contrasting pH conditions that exist in the human stomach (∼pH 2), the intestine (∼pH 7) and the way in which flocculation and dispersion vary according to pH. In food science, the term “coagulation” is analogous to the use of “flocculation” by soil scientists.

For example, most clay minerals flocculate at an acidic pH and some, such as kaolinite, even at circum-neutral pH values. Dispersion of clay minerals usually requires alkaline pH values, although the presence of salts and polymers of various kinds may encourage flocculation even at alkaline pH values. If the pica substance is protective as a lining of the intestine, as the protective hypothesis suggests, we would expect the substance to flocculate in the intestine (∼pH 7).

No specific procedure is suggested here, but where it is hypothesized that a pica substance acts as a coating or lining, it would be important to determine dispersion/flocculation characteristics in the appropriate physiological environment. Visual inspection of how fine-grained geophagic materials behave when dispersed in simulated stomach or intestinal conditions adjusted to appropriate pH values would be an appropriate first step in evaluation of flocculation.

To further determine the plausibility of a coating mechanism, the affinity of pica substances for binding with mucus could be tested. There is evidence that both clay and starch exert some protective action against damage to the gut mucosa by proteolytic enzymes [98] and may reduce inflammation induced by antigens [44]. Mechanisms for these effects are unknown, but may involve binding of harmful substances by the pica substance thereby preventing the harmful material from reaching the epithelial cells. Alternatively, pica substances may directly modify cytokine production by mucosal cells, as appears to be the case with diosmectite. Cytokines are involved in the inflammatory response, so altering their release may reduce inflammation. Appropriate *in vitro* techniques [44], [99] and *in vivo* models in rodents [100], [101] have been described elsewhere.

### 4. Mineralogical Analyses

Although early articles describing geophagic earth do contain useful observations about color, plasticity and the behavior of those who ate it e.g. [102]–[108], they are not informative about mineralogy, since the nature of clays was only elucidated in the 1930's with the application of X-ray diffraction (XRD) techniques.

In brief, XRD revealed that clays were generally made up of crystalline minerals with a layer structure, the so-called clay minerals. Most clay minerals are hydrous or hydroxylated alumino-silicates and comprise two fundamental units, namely a tetrahedral and an octahedral sheet. These names refer to the fact that the structural cations are co-ordinated in a four-fold tetrahedral manner or six-fold octahedral manner by oxygen or hydroxyl anions.

The basic tetrahedral building block is the SiO_4_^−4^ silicon tetrahedron in which three oxygens are linked through their apices (not the faces or edges) to adjacent tetrahedra to form sheets of continuous six-membered rings of tetrahedral, in which the unshared oxygens all point in the same direction. Thus, one side of a tetrahedral sheet consists of a hexagonal network of shared oxygens, whilst the other side is formed by the remaining so-called ‘apical’ oxygens. The formula for the tetrahedral sheets is Si_4_O_10_^−4^. Tetrahedral co-ordination usually accommodates Si^4+^ and Al^3+^.

The octahedral building block can be viewed as consisting of 2 planes of closely packed oxygens or hydroxyls, consisting of 8-sided polyhedra (octahedra) where the edges of the octahedra are linked to give a hexagonal pattern. In the center of such a sheet and adjacent to every anion, there are three octahedral sites which may be occupied by cations such as Al^3+^, Fe^3+^, Fe^2+^ and Mg^2+^, each cation being surrounded by six anions. Thus, the octahedra coordinate trivalent and divalent cations with formula such as Al_2_(OH)_6_ or Mg_3_(OH)_6_ so that the octahedral unit varies according to cation occupancy.

By joining tetrahedral and octahedral sheets together, two basic clay mineral units known as layers can be formed. The unit formed by linking one tetrahedral sheet and one octahedral sheet together is called a 1∶1 layer. A tetrahedral sheet can be similarly linked to the other side of an octahedral sheet, and the unit formed is known as a 2∶1 layer. Substitutions of cations by others of lower valence such as Al^3+^ for Si^4+^ in tetrahedral sheets, and Mg^2+^ or Fe^2+^ for Al^3+^ in octahedral sheets are commonplace, thus yielding an overall net negative charge. This charge may be neutralised by fixed cations, hydrated exchangeable cations, or by octahedrally coordinated hydroxyl groups or sheets, all of which occupy a position between the layers known as the interlayer. Because of their fine particle size, clay minerals are generally considered to be the most active of the mineral components in soils and sediments. Of course, their properties and behavior vary according to details of their chemistry and structure, particularly at the surfaces of the particles. Mineralogy is thus relevant to the nutritional and protective hypotheses of geophagy and for example may indicate whether a clay will bind with dietary micronutrients like Fe or Zn or if it is capable of adsorbing toxic chemicals, viruses, or bacteria.

X-ray diffraction is the primary method for determining the mineral composition of clays, although other methods such as infrared spectroscopy, thermal analysis and scanning electron microscopy provide useful complementary information. For geophagic materials, a bulk sample should be analyzed by XRD to establish total quantitative mineralogy and relative amounts of clay and non-clay minerals. A convenient method is the Reference Intensity Method [109], [110] which has been applied to analysis of clay materials. For quantitative analysis of these materials it is essential that the sample is presented to the diffractometer in a completely randomly oriented form, and the spray drying technique developed by Hillier [111] is the best way of ensuring this.

Characterization of clay minerals requires that they are first separated from the larger, non-clay fractions after dispersion in deionized water. Then, slides for the diffractometer must be prepared from the aqueous clay suspensions. In contrast to the quantitative analysis of the bulk fraction, characterization of the clay minerals by XRD is most effectively done by ensuring that they are presented to the diffractometer in an oriented way. This enhances the intensities of the diagnostic basal reflections from the layered clay structure, thus enabling the specific minerals to be easily identified [112]. Oriented clay aggregates can be easily prepared using various techniques, including drying down onto glass slides or a filter peel technique. Nevertheless, unequivocal identification of certain clay minerals requires the use of supplementary treatments. Smectite minerals are identified by the use of glycerol or ethylene glycol which expands their layer structure in a characteristic way [113]. The determination of halloysite, as opposed to kaolinite, can be done through the rapid formation of an intercalation complex with formamide [114]. Mixed layer minerals can be identified through a comparison of calculated and observed diffraction curves [115]. The presence of aluminous interlayers in expansible clay minerals can be established by heat treatments showing that the contraction of the layer spacing, which is usually observed when the clay mineral is in the hydrated state, is effectively inhibited.

Scanning electron microscopy is a useful accessory in the characterization of the mineralogy of geophagic soils and has the additional advantage that the original texture and fabric of the material being examined is maintained. In contrast, XRD examination of geophagic materials destroys such original structural elements because it requires samples to be prepared by particle size separation or homogenization by grinding. Again, if the instrument is equipped with micro-analytical facilities then it is possible to determine with which mineral the elements of interest, such as Zn and Fe, are associated. Such an association does not indicate the bioavailability of these elements, although this may possibly be inferred from knowledge of the susceptibility of particular minerals to decomposition during weathering. For example, if iron is associated with a mineral which is resistant to weathering such as tourmaline, then it would be reasonable to suppose that the element would not be bioavailable. However, if iron is associated with ferromagnesian minerals such as olivine or biotite, both of which are susceptible to rapid decomposition during weathering, then it is more likely that the element could be bioavailable.

### 5. Chemical analyses

#### 1. Total Elemental Composition Analysis

Determining the presence of any nutritionally relevant element is of obvious significance in the context of hypotheses relating geophagy to micronutrient deficiency.

Analysis of the total elemental composition of pica substances and their controls may be performed by a variety of techniques including X-ray fluorescence (XRF), inductively coupled plasma–atomic emission spectroscopy (ICP-AES), and instrumental neutron activation analysis (INAA). It is important to bear in mind that although a total chemical analysis provides useful background information about the presence of nutrients and other substances in pica samples, it is of limited use , by itself and in fact may be positively misleading if not accompanied by an assessment of bioavailability to the consumer (cf. *Bioavailability* section, below).

ICP-AES is the most widely used method for mineral analyses in foods and would be appropriate for the analysis of uncooked foods, ice, and freezer frost [116]. Atomic absorption (AA) instruments may also be used, although inductively coupled plasma-atomic emission spectrometers are preferred if available, because they are capable of measuring multiple elements on a single sample in a single run [116]. In contrast, neither ICP-AES nor AA is recommended for rock or mineral samples because the sample must be prepared in solution form prior to analysis. If the sample contains resistant minerals, it is often difficult to ensure that a rock or soil sample has been totally dissolved.

XRF analysis is recommended as the analytical method of choice for inorganic materials because the instrumentation is widely available and has become the standard method for the analysis of major and trace elements of rocks following the procedures developed by Norrish and Hutton [117] and Leake et al [118]. XRF analysis is performed on pressed-powder discs and involves no pre-treatment other than a simple crushing procedure.

In principle, preparation is not difficult with INAA, and indeed this method is specially recommended by Mahaney and Krishnamani for the analysis of geophagic soils [3]. However, the technique requires specialized irradiation facilities usually associated with nuclear reactors. For this reason, it is not as widely available as XRF analysis, and in any case possesses no special advantages over XRF in the determination of major and trace elements that are likely to be of biological importance.

#### 2. NaCl content

Pica, specifically geophagy, was once attributed to the physiological requirement for NaCl [119], but this hypothesis has more or less been ruled out as a motivation for human pica following the establishment of the salt-deficient nature of most geophagic materials. Therefore, determination of NaCl content may be considered to be of lesser importance.

The total soluble sodium and chloride content of non-food substances may be measured in a water extract by ICP-AES, flame photometry or ion exchange chromatography [120]. ICP- AES may be the most convenient if it is already being used to determine total elemental composition (see above). For all of these tests, a correction must be made for exchangeable sodium which should be determined separately; non-exchangeable NaCl may not be detectable although it may be available to humans.

The total quantities of soluble salts in soils may be determined by electrical conductivity (EC) measurements which are usually performed on soil∶water mixtures in a 1∶2.5 ratio. Values of EC are usually given in mS cm^−1^ at 25°C. For purposes of predicting the impact of salinity on crop yields, EC values from soils of 0–2 are described as salt-free, 4–8 as slightly saline, 8–15 as moderately saline and >15 as strongly saline [120].

#### 3. Measurement of pH and buffering capacity

As food enters the stomach, the pH of the stomach contents (digesta) rises to approximately the pH of the ingested food. This stimulates gastric acid secretion, causing the pH of the digesta to gradually decline. As the pH decreases, many nutrients, e.g. Fe, become more soluble. This increase in solubility favors subsequent absorption in the small intestine. The absorption of iron and possibly other nutrients is impaired in people with low gastric acid secretion [121], presumably because the release of nutrients from foods into soluble forms is reduced. Solubility in the duodenum is a key factor affecting iron absorption. Because iron is much more soluble at low pH than at neutral pH, it follows that iron absorption will be impaired by consumption of substances that may buffer stomach acid and thereby prevent the pH from going as low as it otherwise would. For example, calcium carbonate, a widely used antacid, depresses iron absorption in rats [122]. For these reasons, it is important to measure the pH and buffering capacity of pica substances.

Soil pH measurements are typically made by an electrometric method using glass-calomel electrodes on soil suspensions in a soil∶water ratio of 1∶2.5. The use of 0.01 M CaCl2 suspensions may also be used, but the pH values obtained are typically 0.5–0.9 units below the values obtained for water. A detailed account of the full procedure which is applicable to agricultural soils is found in Peech [123].

For pica substances it may also be important to measure pH under conditions that reflect preparation techniques. For example, if the sample is chewed or ground before consumption (rather than swallowed whole, like a pill), then it may be important to measure pH immediately after grinding for the period of time specified in the oral interview. Such a measurement is known as “abrasion pH” and can yield surprising results. For example, Grant found an abrasion pH of 9.3 for fresh Stone Mountain granite after grinding it in distilled water for 2.5 minutes, and compared this with pH values of 5.8 to 7.0 found for well water which had equilibrated with fresh granite at depth [124]. Measurement of the pH of geophagic materials is relevant to the hypotheses that micronutrient deficiency is a cause of pica and that anemia is a consequence, since intraluminal pH affects iron absorption. A change in pH may also affect the growth of harmful microbes and parasites; lower pH likely inhibits their proliferation.

Buffering capacity is a better indicator of the impact of an ingested substance on luminal pH in the stomach and proximal small intestine than the pH of the substance. Buffering capacity may be defined as the number of moles of a strong acid or base required to change the pH of a given quantity of a buffer by 1 pH unit [125, p. 123]. If a material has a high buffering capacity, it will tend to neutralize stomach acid as it is secreted, and this will prevent the pH of luminal contents from falling as far during the gastric phase of digestion. The pH of duodenal contents is influenced by the pH of digesta emptying from the stomach; the lower the pH of stomach contents, the lower the pH in the duodenal lumen.

The buffering capacity of soils is directly related to pH, organic matter content and soil type[126]. The importance of the mineralogy of geophagic soils in effectively buffering against excess acidity in the digestive tract was discussed by Wilson [127].

#### 4. Cation exchange capacity

Cation exchange capacity (CEC) is a measure of how readily a substance can exchange adsorbed cations with cations in a surrounding solution, and may therefore be relevant to an overall assessment of the activity of a geophagic material with respect to adsorption. It has been suggested that pica substances can adsorb dietary Fe and Zn [54], thereby causing anemia, and that they can bind or adsorb harmful pathogens and chemicals, thus offering protection [35], [36], [45]. The CEC of clay minerals may be related to their ability to act as sources or sinks of macro/micro nutrients and also to cytoprotection [54]. It should be noted, however, that adsorption and binding may involve mechanisms other than cation exchange.

Cation exchange in soils can be either pH-independent or pH-dependent. The former category is related to permanent charge as a result of isomorphous substitution by lower valence cations into the structure of clay minerals such as montmorillonite. In contrast, pH-dependent binding mainly relates to variable charged edge sites of 1∶1 clay minerals like kaolinite. These sites become negatively charged at pH values above approximately 5, depending on the provenance of the kaolinite, and become positively charged at lower pH values, thus conferring an anion exchange capacity to these soils. For variable charge soils, CEC should be measured in a 1 M KCl extract at the unbuffered pH of the soil. For soils of ∼ neutral pH which are not saline or calcareous, CEC may be measured in an ammonium acetate extract adjusted to pH 7 [128].

#### 5. Organic carbon

Organic matter may act as a source of N, P or S, (although the actual form in which these nutrients are held is almost always unspecified). It may also increase the soil CEC and overall adsorption capacity. It should be noted, however, that most geophagic soils contain little to no organic matter. Most routine determinations of organic carbon in soils are made by the Walkley-Black dichromate method [128] or by dry combustion. It should be noted that the Walkely-Black method does not determine carbon that is present in the form of charcoal, which in some soils may form a substantial proportion of the total organic carbon. Sometimes organic carbon is multiplied by a conversion factor of 1.72 to give the percent organic matter [128].

#### 6. Determination of labile forms of iron oxide in soils

The hypothesis that geophagic soils and clays are consumed because they act as a supplementary source of iron necessitates an assessment of the amount of labile or active iron that these materials contain. Determination of total iron (cf. *Total Elemental Composition*) is of little relevance in this respect. A standard method in soil science which may be useful in the context of geophagic materials is the determination of the ratio of oxalate soluble iron (Fe_o_) to dithionite soluble iron (Fe_d_). Acid ammonium oxalate is used to extract fine-grained, poorly crystalline iron oxides which may be assumed to be the most labile. Dithionite-citrate-bicarbonate treatment extracts practically all secondary iron oxides, including highly crystalline forms, without differentiating the mineral phases [129].

### 6. Biological interactions

#### 1. Bioavailability

Bioavailabilty may be defined as the proportion of an ingested nutrient that is absorbed and either utilized in a metabolic pathway or sequestered in body stores [130]. The majority of the analyses of pica materials measure total mineral concentrations but do not attempt to assess bioavailability. This approach almost certainly overestimates the content of bioavailable nutrients and “is analogous to an agronomist assessing the ability of a soil to grow crops on the basis of the total nutrient mineral content of the soil without considering what is available for uptake by the crop” [127].

A variety of *in vitro* and *in vivo* methods have been developed and used to assess the bioavailability of iron from foods, dietary supplements and fortificants. Perhaps the simplest *in vitro* method is to mix the food with water, adjust the pH to 2 (approximate gastric pH), incubate with agitation for a period of time, centrifuge, and measure the concentration of the iron in the supernatant. Solubility measured in this way may be considered as a relative index of iron bioavailabilty, since iron must be soluble to be taken up by intestinal mucosal cells. This rather crude approach has been refined to better reflect conditions within the gastrointestinal tract. In one such method [131], food or meal samples are blended in water, adjusted to pH 2, incubated in the presence of pepsin (a gastric protease), and incubated in the presence of pancreatin (digestive enzymes isolated from pancreatic secretions), bile salts, and a dialysis bag containing a buffer that gradually raises the pH to between 6 and 7. Iron that is solubilized during the pancreatin phase of the simulated digestion dialyzes into the dialysis bag. The concentration of this “dialyzable iron” is considered to be a predictor of iron bioavailability.

This dialyzabile iron method described above has been further modified to include measurement of the uptake of the dialyzable iron by cells grown in culture [132]. Briefly, foods or meals are suspended in isotonic saline solution, adjusted to pH 2, and incubated in the presence of pepsin for 1 hour. The pH is then adjusted to between 6 and 7, pancreatic digestive enzymes are added, and the mixture is placed in an upper chamber situated in a six-well plate with a confluent monolayer of Caco-2 cells growing on the bottom. The upper chamber containing the digesta and the pancreatin/bile mixture is separated from the growth medium in the lower chamber by a semipermeable membrane. The plates are incubated with gentle rocking for an additional 2 hours. Iron that is dialyzable into the bottom chamber and bioavailable is taken up by the Caco-2 cells. After this incubation, the upper chamber is removed and the cells are incubated for an additional 22 hours to allow time for the cells to produce ferritin, an iron storage protein that is synthesized intracellularly in response to increased intracellular iron concentrations. After this incubation is complete, the cells are harvested and ferritin is measured using an immuno-radiometric method. The concentration of ferritin in the cells is expressed as ng ferritin/mg cell protein.

Only four studies have assessed the bioavailability of minerals in geophagic materials using *in vitro* techniques. The clays that Johns and Duquette analyzed released Ca, Cu, Fe, Mg, Mn and Zn into solution in biologically significant amounts following extraction with tannic acid adjusted to pH 2 with HCl in an electrolyte solution of 0.1 mol NaCl/L [36]. Two have used a physiologically-based extraction test (PBET) to determine the potential nutrients that the sample could contribute to the consumer [133], [134]. This technique is a much closer approximation of the human digestive system than the total acid digests that typically have been performed. The fourth study attempted to mimic conditions of the gut by looking at the nutrients that geophagic materials could contribute, as well as at their capacity to *bind* nutrients, thus rendering them unavailable [54], [135]. This is the first and only use of this method. Results indicated that the five geophagic samples analyzed could contribute bioavailable calcium but significantly reduced the availability of iron and zinc in the diet. Clearly, more studies of bioavailability are necessary.

The Caco-2 model has yet to be used in any pica studies, although it is a well-established method in food science for realistic approximation of *in vivo* bioavailability [136]. It seems to be a very promising way to establish bioavailability of micronutrients in any pica substances, e.g. starch, uncooked rice, charcoal, and baby powder. Thus, to establish bioavailabilty of micronutrients in pica substances, we recommend the use of the Caco-2 model. In addition, the Caco-2 model could be used in the analysis of available micronutrients in foods in the presence and absence of pica substances, indicating potential effects that pica may have on the availability of micronutrients in foods.

In food and nutritional sciences, animal models are also widely used to assess bioavailability. One such model is the piglet hemoglobin repletion model [137]–[139]. In this model, iron deficient anemic piglets are fed diets containing the iron source of interest. Iron intakes are carefully monitored and hemoglobin concentrations are measured at the beginning and end of the feeding period. Hemoglobin iron gain over the feeding period is calculated from changes in hemoglobin concentrations and blood volume. Iron absorption is calculated by dividing the hemoglobin iron gain by iron intake. The piglets are useful for assessing iron bioavailability from human foods because their gastrointestinal tracts are similar to humans and they readily consume human foods.

Iron bioavailability from foods may also be determined in experiments with human subjects. Human studies are preferred over animal models but are expensive and time consuming. The most widely used human methods require the use of radio or stable isotope tracers. Briefly, foods are labeled with an iron isotope either intrinsically by growing the food hydroponically in solutions containing the tracer or extrinsically by mixing a solution of the tracer with the food. The labeled food is then fed to the subjects following an overnight fast. After two weeks during which time the absorbed tracer is incorporated into hemoglobin, a blood sample is drawn and analyzed for the tracer. Iron absorption is calculated as the proportion of the ingested tracer present in the blood as hemoglobin iron.

A small number of *in vivo* pica studies have been carried out, most of which used a rodent model [140]–[146]. Several small studies of pica and micronutrient absorption in humans have also been performed [55], [147]–[150]. Some of these studies showed that pica substances decrease iron absorption, while others found no effect. Because their protocols have varied immensely, it is difficult to interpret the conflicting results. Our understanding of the consequences of the consumption of pica substances would be enhanced through the consistent use of the Caco-2 cell model as well as appropriate *in vivo* studies in pigs and humans.

#### 2. Protection

Evidence is beginning to accumulate that pica substances are able to reduce the harmful effects of chemicals and pathogens by adsorbing them. At this point, it is known that substances that those who engage in pica consume have the capacity to bind a variety of materials, including pharmaceuticals [151]–[156], poisons [48], [157]–[159], bacteria [99], [160], and viruses [161]–[163]. There are very few studies where actual pica materials were used to evaluate their detoxifying capacity. Instead, these studies were conducted with pure substances (clays, charcoal, starch, etc) purchased from scientific supply companies instead of substances provided by pica consumers. Those that analyzed the detoxification capacity of substances that were actually consumed primarily concerned pica-like substances consumed by animals [164]–[168].

The techniques used by those studying the detoxifying capacity of soils eaten by non-human animals are appropriate for those eaten by humans, i.e. the determination the adsorptive maxima. Yet only two groups have analyzed the capacity of human geophagic soils to bind potentially harmful substances [36], [45], [169]. Johns and Duquette measured the capacity of geophagic clays, mainly of kaolinitic and/or smectitic composition, to adsorb tannic acid, a plant secondary compound harmful in large quantities. They found that although most of the clays tested did indeed significantly adsorb tannic acid, it did not occur to an extent necessary to reach non-toxic levels if the clays were consumed directly with unprocessed plant food. It was concluded that clays were consumed with plant foods containing tannins because these foods were made more palatable by the clays.

Dominy et al tested the detoxifying capacity of kaolin using the much more complicated TNO Intestinal Model built by TNO Life Sciences, the Netherlands (http://www.tno.nl/content.cfmcontextmarktencontentproductlaag1195laag2320item_id1100Taal2). They found that kaolin (commercial sample) reduced the availability of two types of tannins and quinine (another plant secondary compound). Unfortunately, the TNO model is primarily used to test emerging pharmaceuticals and is therefore not readily accessible for academic research.

Our understanding of the detoxifying effects of pica is not complete if we look solely at the potential to detoxify plant secondary compounds or even other chemicals broadly speaking. Studies are needed in which the pathogen binding capacity of human geophagic clays is evaluated, i.e. viruses, bacteria. For appropriate protocols, we can look to those studies that tested the capacity of pure clays, starches and charcoals to bind bacteria and viruses [99], [160]–[163]. However, these methods will likely require some modification.

#### 3. Parasitology

If pica substances are a vector for nematode infection, this could explain the relationship between pica and anemia [88]. The protocol that we have used to examine the parasitological profile of pica substances (earth, chalk, charcoal preserved in formalin) was as follows: The entirety of the sample was passed through a double layer of 1-mm steel mesh into a Petri dish. Water was then added to the dish. When there was too much sediment to accurately read a dish, the sample was then split into halves or quarters and the examination was repeated until the entire sample was examined. The entire dish was scanned using an inverted microscope with a mechanical stage, and a 10×-objective. Eggs and larvae were confirmed and speciated using a 40×-objective. This protocol, when applied to stool samples where low concentrations of eggs and larvae are present, has proven to have greater or equal sensitivity than the sugar and flotation techniques commonly used to examine parasitic stages in soil [170].

## Discussion

This paper has outlined six groups of procedures and methodologies spanning the social, soil, food and nutritional sciences. Together, these constitute a comprehensive approach to the study of pica that can generate data to test the many hypotheses about its causes and consequences. We are certain that this approach can be further expanded and improved, and we encourage all addendums, comments, and criticisms to be actively communicated through the PloS portal, to facilitate a more rapid understanding of this enigmatic consumption behavior.
